# Superior Aortic Recess Mimicking Takayasu Arteritis in a Patient With Adult-Onset Still’s Disease

**DOI:** 10.7759/cureus.91000

**Published:** 2025-08-25

**Authors:** Yoshinori Noguchi, Yasuhiro Hata, Tomoki Kiyama, Satoshi Ando, Yasuhiro Osugi

**Affiliations:** 1 General Practice, Toyota Regional Medical Center, Toyota, JPN; 2 General Internal Medicine, Fujita Health University Okazaki Medical Center, Okazaki, JPN; 3 General Practice, Minami Seikyo Hospital, Nagoya, JPN; 4 Community Medicine, Fujita Health University, Toyoake, JPN

**Keywords:** adult-onset still's disease, aortic wall thickening, fuo, superior aortic recess, takayasu arteritis

## Abstract

The superior aortic recess, a normal pericardial extension around the ascending aorta, can be misinterpreted as pathological findings on imaging studies, potentially leading to misdiagnosis of conditions such as Takayasu arteritis. We report a case of persistent fever and joint pain initially suspected of having Takayasu arteritis based on contrast-enhanced CT showing apparent aortic wall thickening. Laboratory tests showed elevated inflammatory markers (CRP: 7.07 mg/dL, WBC: 11,200/μL), microcytic anemia (hemoglobin: 7.8 g/dL), and thrombocytosis (platelets: 599,000/μL). Initial treatment with nonsteroidal anti-inflammatory drugs, sulfasalazine, and low-dose prednisolone was ineffective. Although contrast-enhanced CT suggested aortic wall thickening, a fluorodeoxyglucose positron emission tomography scan/CT revealed no uptake in this area, making a diagnosis of Takayasu arteritis unlikely. Radiological reassessment identified the structure as the superior aortic recess rather than aortic wall thickening, and the patient fulfilled Yamaguchi's criteria for adult-onset Still's disease with markedly elevated ferritin (3,459 ng/mL). Treatment with prednisolone 60 mg daily and subsequent addition of tocilizumab 480 mg weekly led to complete symptom resolution and normalization of laboratory parameters. This case highlights the importance of recognizing normal anatomical variants in radiological interpretation to avoid misdiagnosis, emphasizing the need for comprehensive diagnostic approaches incorporating clinical, laboratory, and appropriate imaging findings to distinguish between vascular pathologies and normal anatomical variants.

## Introduction

Fever of unknown origin remains a challenging clinical problem, with large vessel vasculitis such as Takayasu arteritis representing an important differential diagnosis. Takayasu arteritis is a chronic large-vessel vasculitis that predominantly involves the aorta and its major branches. Its diagnosis relies fundamentally on vascular imaging, with particular emphasis on the detection of aortic wall inflammation, including wall thickening and luminal changes [1]. However, normal anatomical structures can sometimes mimic pathological findings on cross-sectional imaging. The superior aortic recess is a normal posterior extension of the pericardial cavity surrounding the ascending aorta, which may be misinterpreted on CT imaging as a mediastinal mass or lymphadenopathy [2]. We report a case where apparent aortic wall thickening on CT initially suggested Takayasu arteritis in a patient with fever of unknown origin but was ultimately identified as the superior aortic recess in a patient diagnosed with adult-onset Still's disease (AOSD).

## Case presentation

A 44-year-old female presented to our clinic with a seven-month history of persistent fever, bilateral finger joint pain scattered across the distal and proximal interphalangeal as well as metacarpophalangeal joints, and fatigue.

The patient had experienced continuous fever around 38°C for seven months. Four months prior to the presentation, she developed finger joint pain and consulted another physician. The referring physician diagnosed her with limited systemic sclerosis based on positive anti-centromere antibody results and started her on nonsteroidal anti-inflammatory drugs. Despite treatment, she continued to experience daily fevers above 38°C and developed generalized myalgia. Sulfasalazine and prednisolone 10 mg were added to her regimen without improvement, and she was subsequently referred to our department.

At initial examination, her vital signs were temperature 37.1°C, blood pressure 104/67 mmHg, pulse 104 beats/minute, and respiratory rate 16 breaths/minute. Physical examination revealed only faint erythema on both upper arms, lower back, and both thighs. There was no cervical lymphadenopathy, temporal artery tenderness, finger joint swelling, or skin sclerosis. No blood pressure difference was observed between the upper limbs, and no vascular bruits or pulse deficits were noted in the upper extremities. Laboratory tests showed aspartate aminotransferase 51 U/L, alanine aminotransferase 30 U/L, lactate dehydrogenase 440 U/L, C-reactive protein 7.07 mg/dL, white blood cells 11,200/μL (neutrophils 9,700/μL), red blood cells 3.90×10^6/μL, hemoglobin 7.8 g/dL, hematocrit 27.7%, mean corpuscular volume 71.0 fL, and platelets 599,000/μL, indicating inflammation, microcytic anemia, and mild liver dysfunction. Autoimmune screening revealed positive antinuclear antibodies (1:640, speckled pattern) and anti-centromere antibodies (107 U/mL), while rheumatoid factor, anti-CCP antibodies, anti-SS-A, anti-SS-B, anti-ARS, anti-RNA polymerase III antibodies, MPO-ANCA, and PR3-ANCA were negative. Two sets of blood cultures were negative. The results of the blood tests are summarized in Table 1.

**Table 1:** Blood tests on admission mEq/L: milliequivalents per liter, g/dL: grams per deciliter, mg/dL: milligrams per deciliter, ng/mL: nanograms per milliliter, /µL: per microliter, U/L: units per liter, U/mL: units per milliliter, IU/mL: international units per milliliter, fL: femtoliter, Ig: Immunoglobulin, RF: rheumatoid factor, CCP: cyclic citrullinated peptide, ANA: antinuclear antibody, SS-A: Sjögren’s syndrome type A, SS-B: Sjögren’s syndrome type B, ARS: aminoacyl-tRNA synthetase, RNA polymerase III: ribonucleic acid polymerase III, MPO-ANCA: myeloperoxidase-anti-neutrophil cytoplasmic antibody, PR3-ANCA: proteinase 3-anti-neutrophil cytoplasmic antibody

Laboratory item/reference range	Result
Sodium (138-145 mEq/L)	141
Potassium (3.6-4.8 mEq/L)	4
Chloride (101-108 mEq/L)	103
Total bilirubin (0.4-1.5 mg/dL)	0.2
Aspartate aminotransferase (13-30 U/L)	51
Alanine aminotransferase (7-23 U/L)	30
Lactate dehydrogenase (124-222 U/L)	440
Alkaline phosphatase (38-113 U/L)	80
Total protein (6.6-8.1 g/dL)	7.1
Albumin (4.1-5.1 g/dL)	3
Blood urea nitrogen (8-20 mg/dL)	10.4
Creatinine (0.46-0.79 mg/dL)	0.77
C-reactive protein (0.00-0.14 mg/dL)	7.07
Ferritin (10-60ng/mL)	3459
White blood cells (3.3-8.6 /µL)	11.2
Segmented neutrophils (42.4-75.0 /µL)	97
Lymphocytes (18.2-47.7 /µL)	104
Monocytes (3.3-9.0 /µL)	3.3
Eosinophils (0.4-8.6 /µL)	20
Basophils (0.2-1.4 /µL)	1
Red blood cells (3.86-4.92×10^6/µL)	3.9
Hemoglobin (11.6-14.8 g/dL)	7.8
Hematocrit (35.1-44.4 %)	27.7
Mean corpuscular volume (83.6-98.2 fL)	71
Platelets (15.8-34.8×10^4/µL)	59.9
IgG (861-1747 mg/dL)	1754
IgM (50-269 mg/dL)	171
IgA (93-393 mg/dL)	269
IgG4 (11-121 mg/dL)	30.9
Rheumatoid factor (<15 IU/mL)	<15
Anti-CCP antibody (<4.5 U/mL)	<0.6
Anti-nuclear antibody (<1:40)	1:640 speckled pattern
Anti-centromere antibody (<10 U/mL)	107
Anti-SS-A antibodies (<10 U/mL)	<10
Anti-SS-B antibodies (<10 U/mL)	<10
Anti-ARS antibody ( <10 U/mL)	<10
Anti-RNA polymerase III antibody (<28 U/mL)	<28
MPO-ANCA (<3.5 U/mL)	<0.6
PR3-ANCA (<3.5 (U/mL)	<0.6

**Figure 1:**
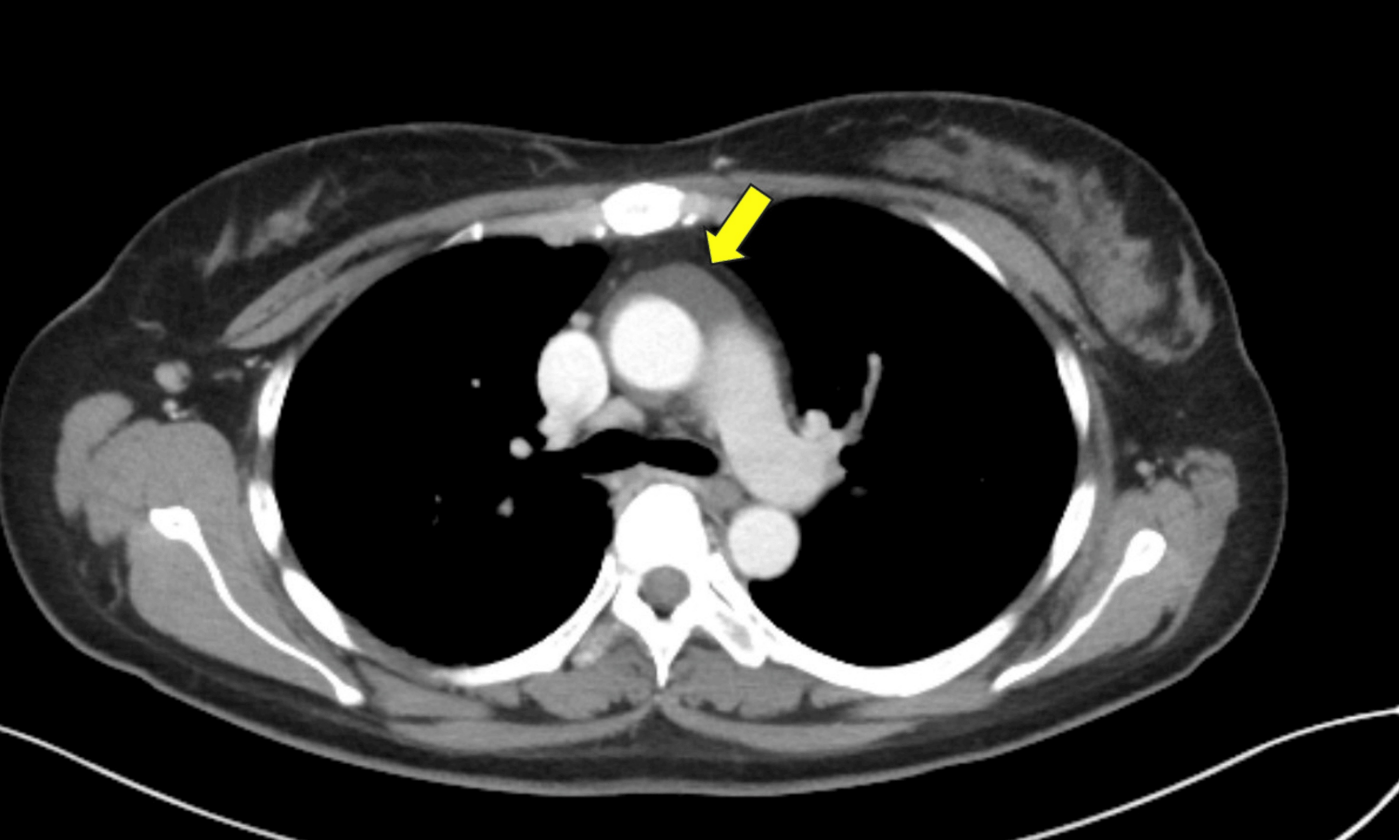
Contrast-enhanced CT Apparent wall thickening was observed at the base of the ascending aorta (arrow). CT: computed tomography

**Figure 2:**
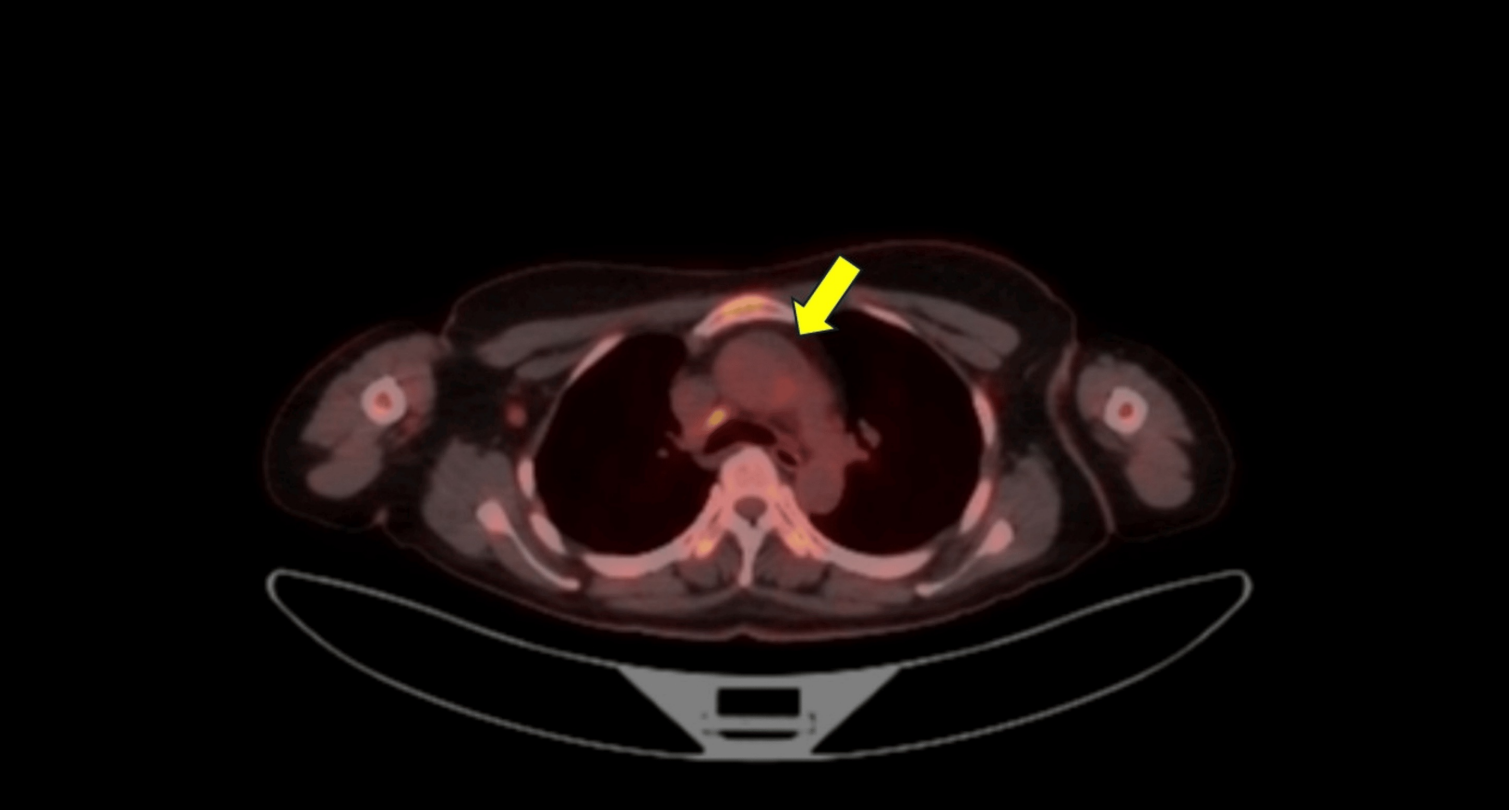
Axial view of FDG-PET/CT FDG-PET/CT showed no uptake in the area of the ascending aorta where wall thickening was observed on CT (arrow). FDG-PET/CT: fluorodeoxyglucose positron emission tomography/computed tomography

**Figure 3:**
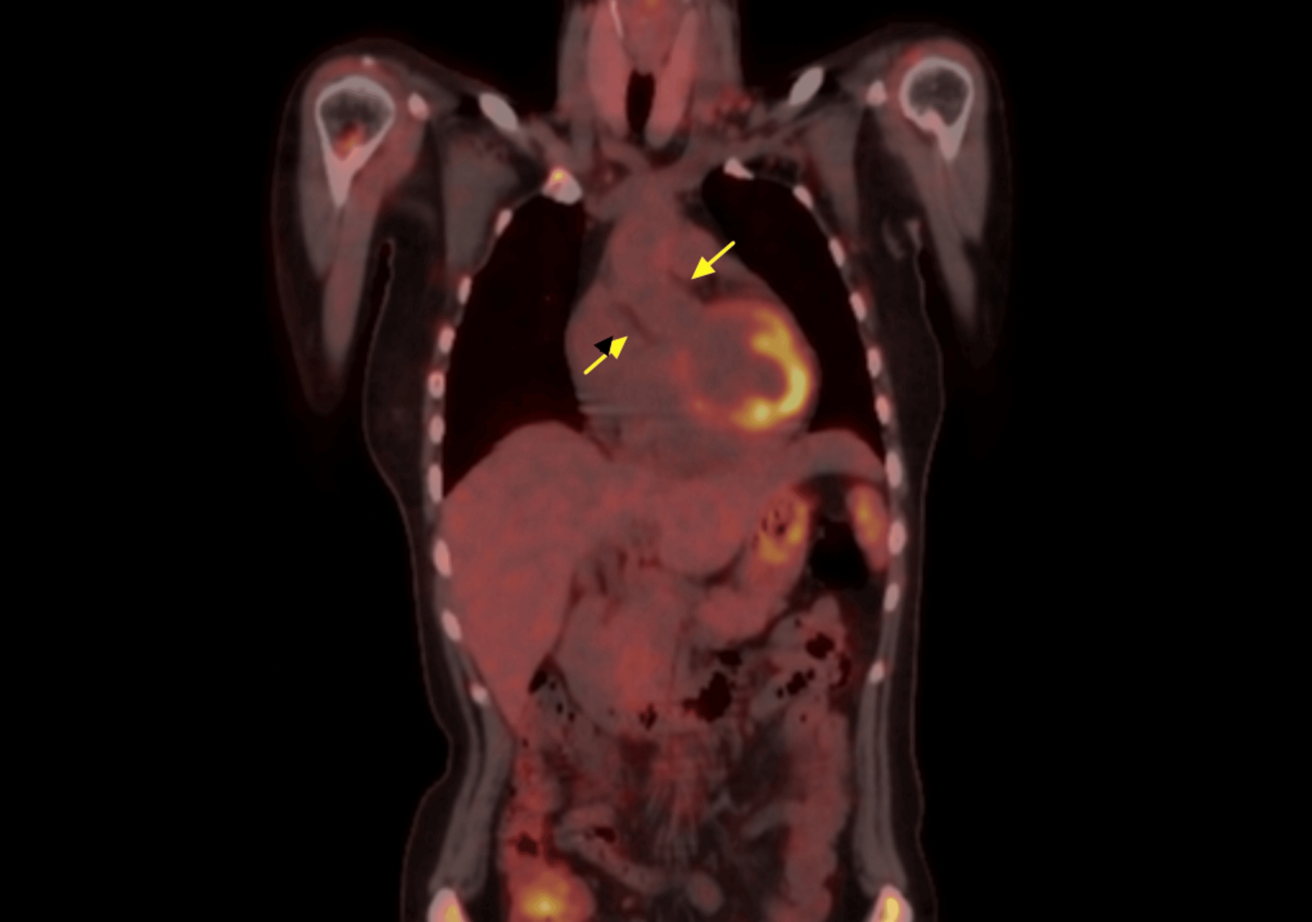
Coronal view of FDG-PET/CT No FDG uptake was observed in the wall of the ascending aorta or in any other arteries on PET/CT (arrow). FDG: fluorodeoxyglucose, PET/CT: positron emission tomography/computed tomography

Contrast-enhanced CT showed apparent wall thickening of the ascending aortic root, suggesting Takayasu arteritis (Figure 1). However, fluorodeoxyglucose positron emission tomography/computed tomography (FDG-PET/CT) showed no uptake in this area, making this diagnosis unlikely (Figures 2-3).

During the clinical course, the patient developed a sore throat, and her ferritin level was markedly elevated at 3,459 ng/mL. Based on established diagnostic criteria for AOSD, which are discussed in detail in the Discussion section, the patient’s presentation was considered consistent with the diagnosis of AOSD. Treatment with prednisolone 60 mg (approximately 1 mg/kg/day) was initiated, resulting in prompt defervescence and reduction in CRP and ferritin levels. When prednisolone was tapered to 40 mg/day, fever and joint symptoms recurred, necessitating an increase back to 60 mg/day. Tocilizumab 480 mg weekly was added, leading to improvement in symptoms and laboratory findings. Subsequently, prednisolone was gradually tapered without symptom recurrence.

## Discussion

This case involved a relatively young woman meeting the criteria for classical fever of unknown origin with CT findings of apparent aortic wall thickening. The patient initially met the two absolute requirements of the 2022 American College of Rheumatology/European Alliance of Associations for Rheumatology Classification Criteria for Takayasu arteritis: age ≤60 years and imaging evidence of large-vessel vasculitis (contrast-enhanced CT showing apparent wall thickening of the ascending aortic root) [1]. However, FDG-PET/CT, which has high diagnostic accuracy for Takayasu arteritis (sensitivity 92.6% in Tezuka's report and 100% in Karapolat's report, with specificity of 91.7% and 88.9%, respectively) [3,4], was negative, excluding this diagnosis. Upon reevaluation of the contrast-enhanced CT images by a radiologist, the lesion was located anterior to the ascending aorta and superior to the aortic root (near the aortic valve and sinuses of Valsalva). Based on its location, extent, and water-like density, this structure was suspected to be the superior aortic recess. The pericardium surrounds the heart and extends upward to cover the main pulmonary artery, ascending aorta, and superior vena cava. The visceral pericardium (epicardium) adheres closely to the heart and great vessels, forming recesses and sinuses. The superior projection is called the superior aortic recess, which is found around the ascending aorta. The superior aortic recess is divided into anterior, posterior, and right lateral parts, with the posterior part specifically called the superior aortic recess. These recesses and sinuses can be visualized on cross-sectional imaging even without pericardial effusion if they contain some fluid, potentially being misinterpreted as lymphadenopathy or masses [2]. In this case, it was misinterpreted as aortic wall thickening.

The patient fulfilled all major criteria of Yamaguchi's classification (fever, arthralgia, rash, leukocytosis with neutrophilia) and two minor criteria (sore throat, liver dysfunction) [5], with markedly elevated ferritin at 3,459 ng/mL. After excluding infections, malignancies, and other rheumatic diseases, AOSD was diagnosed. Yamaguchi's criteria have high diagnostic accuracy with 96.3% sensitivity and 98.9% specificity, and the treatment course was consistent with AOSD [6].

## Conclusions

In this case, a relatively young woman with classical fever of unknown origin presented with imaging findings suggestive of aortic wall thickening, initially raising suspicion for Takayasu arteritis. However, the ultimate diagnosis was AOSD. The superior aortic recess, which can be misinterpreted as lymphadenopathy or masses, was misdiagnosed as aortic wall thickening in this case. This highlights the importance of anatomical knowledge in image interpretation. A comprehensive diagnostic approach incorporating clinical, laboratory, and appropriate imaging findings is essential to distinguish between vascular pathologies and normal anatomical variants.
